# Editorial: Innovative teaching and learning in health education and promotion

**DOI:** 10.3389/fpubh.2025.1770591

**Published:** 2026-01-20

**Authors:** Emília-Isabel Martins Teixeira-da-Costa, María Dolores Ruiz Fernández, Isabel María Fernández Medina, Maria del Mar Jimenez Lasserrotte, Maria Isabel Ventura-Miranda

**Affiliations:** 1 Faculty of Nursing, Universidad de Huelva, Huelva, Spain; 2 School of Health, Nursing Department, University of Algarve, Faro, Portugal; 3 Escola Superior de Enfermagem de Coimbra Unidade de Investigacao em Ciencias da Saude Enfermagem, Coimbra, Portugal; 4 Faculty of Health Sciences, Universidad de Almeria Facultad de Ciencias de la Salud, Almería, Spain

**Keywords:** artificial intelligence in education, digital health education, equity and inclusion, health education, health professions education, innovative teaching and learning

## Introduction

Health education and health promotion are undergoing profound transformation. Demographic transitions, aging populations, increasing multimorbidity, persistent inequities, and rapid technological change are reshaping how learners understand and navigate health. In this evolving context, traditional knowledge-transmission models are no longer sufficient to prepare future professionals for complex, multicultural and digitally mediated environments. Innovation in teaching and learning has therefore become essential, not only to improve learning outcomes, but to strengthen ethical reasoning, equity, and learner autonomy. This global shift echoes recent OECD (1) analyses highlighting how digitalisation, demographic aging and widening social disparities are redefining the competencies required of tomorrow's health workforce and calling for educational approaches attuned to complexity and uncertainty. It is also consistent with the World Health Organization's call for transformative health workforce education, which stresses that conventional training models can no longer meet the demands posed by demographic change, chronic disease burdens, technological acceleration and growing inequities (2).

This Research Topic, *Innovative Teaching and Learning in Health Education and Promotion*, was conceived within this global landscape of change. Its purpose was to bring together diverse contributions that critically reflect on pedagogical innovation across health professions education and community-based health promotion. The collective work highlights not only new tools and strategies, but also the structural and ethical dimensions that shape their implementation.

## Aims and scope of the Research Topic

This Topic includes 40 articles authored by 292 contributors, representing a broad geographical and disciplinary spread. The Topic aimed to explore innovative pedagogical strategies in undergraduate, postgraduate, and lifelong learning; analyse the integration of digital and AI-based technologies into teaching and learning; examine approaches that promote health literacy, behavioral change, and community engagement; foreground inclusive and culturally responsive educational practices and strengthen evidence-based, equity-oriented reform in health education.

These contributions span systematic reviews, meta-analyses, qualitative research, mixed-method studies, implementation reports, and conceptual analyses. Together, they reveal both momentum and tension within the field: between digital acceleration and the persistence of inequities; between technological promise and ethical responsibility; between innovation and the need for rigorous evaluation.

## Critical synthesis of the contributing articles

Across the forty contributions included in this Research Topic, several interrelated thematic patterns emerge, illustrating how innovation in teaching and learning is reshaping health education and health promotion across diverse contexts. While highly viewed articles reflect particular areas of scholarly interest, the collective body of work reveals a dynamic landscape marked by pedagogical reform, digital transformation, equity-oriented approaches, and growing attention to learner wellbeing.

### Evidence-informed and active pedagogical reform

A central theme across the Research Topic is the shift toward evidence-informed, learner-centered pedagogies. Multiple contributions demonstrate that active learning strategies, including problem-based learning, team-based learning, spaced repetition, competition-based learning, case-based approaches, and structured clinical teaching, enhance critical thinking, engagement, professional reasoning, and learner satisfaction across health disciplines, as consistently reported across several empirical and review studies (Su et al.; de Vries et al.; Vagha et al.; Ruan et al.; Li, Tan et al.; Domann et al.; Cortés-Rodriguez et al.).

Complementary evidence from curricular innovations and comparative teaching interventions further reinforces the value of student-centered and participatory approaches in undergraduate and postgraduate education. Studies examining redesigned public health courses, integrated case-based pedagogies and curriculum-level reforms consistently report positive effects on learner engagement, satisfaction and perceived relevance of training (Li, Zheng et al.; Shen, Zhang et al.; Wu et al.; Liu et al.).

However, these contributions also highlight that pedagogical innovation is not merely a matter of adopting new instructional techniques. Its effectiveness depends on tutor preparation, coherent assessment strategies and sustained institutional support, as demonstrated across studies addressing international learning experiences, permanent health education initiatives and collaborative teaching reforms (Albert et al.; Almeida et al.; Zhao et al.). This body of evidence suggests that pedagogical innovation should be understood less as a collection of techniques and more as an institutional commitment to reflective, evidence-informed educational cultures.

### Digital transformation and artificial intelligence in health education

Digitalisation and the integration of artificial intelligence constitute another dominant thematic cluster across the Research Topic. Multiple contributions examine how digital platforms, MOOCs, simulation technologies and AI-supported tools can enhance personalisation, creativity, decision-making and learner autonomy within health professions education, highlighting their potential to transform both teaching processes and learning experiences (Fengling et al.; Mansour and Wong; Wang and Wang; La Mantia et al.).

At the same time, these studies draw attention to persistent and emerging challenges, including digital divides, gaps in digital health literacy, ethical governance, and concerns related to academic integrity and professional reasoning. Evidence addressing these issues underscores that the educational value of digital and AI-supported innovations depends on their responsible integration, supported by ethical frameworks, digital competence development and institutional oversight (Boshnjaku et al.; Taha et al.). Collectively, these studies portray digital innovation in health education as a rapidly evolving field that demands careful stewardship, ethical oversight, and sustained institutional investment.

### Equity, inclusion, and community-engaged learning

Equity, inclusion, and responsiveness to learner and community diversity form a third major theme across the Research Topic. Several contributions demonstrate that structural inequities continue to shape access to meaningful learning opportunities and health promotion, particularly for students with disabilities, culturally diverse learners, and socially marginalized populations, highlighting the need for pedagogical approaches that are inclusive, adaptive and context-sensitive (Liu et al.; Shen, Huang et al.).

Community-engaged, service-learning and faith-based educational approaches are shown to strengthen health literacy, empowerment and participation when aligned with local needs and cultural contexts (Calvo et al.; Bandera-Campos et al.; Reed et al.; Kay et al.; Njarekkattuvalappil et al.). These findings reinforce that educational innovation advances equity only when it engages with social determinants, cultural relevance, and institutional constraints, rather than relying solely on instructional change.

### Digital behaviors, wellbeing, and health literacy

A further cross-cutting theme concerns digital behaviors, learner wellbeing and health literacy. Several contributions examine the dual impact of technology on learning, indicating that while digital tools can enhance engagement and access, excessive or poorly regulated use is associated with increased stress, reduced academic performance and challenges to self-regulation, underscoring the need for balanced and intentional digital integration in educational contexts (Barrientos-Moral et al.).

Contributions addressing digital health literacy, mindfulness education and health behavior change further highlight the importance of embedding metacognitive skills, ethical awareness and self-care within health education curricula. Together, these studies emphasize that promoting learner wellbeing and health literacy is not ancillary but central to sustainable innovation in health education and health promotion (Phan et al.; Deng et al.; Hijazi et al.; Huang et al.; Li, Wu et al.). They highlight the importance of embedding metacognitive skills, ethical awareness and self-care within health education curricula.

### Practice-oriented learning and real-world impact

Finally, several contributions focus on experiential and practice-oriented learning across clinical, community and public health settings. Evidence from studies addressing clinical training, residency education, caregiver education and school-based interventions demonstrates that context-embedded learning supports the development of professional competence, confidence and real-world impact, particularly when learning activities are closely aligned with authentic practice environments and population needs (Ren et al.; Xu et al.; Gil-Hernández et al.; Guo et al.; Li, Li et al.; Shi et al.; Song et al.).

Taken together, the articles within this Research Topic portray a field in dynamic transition. They reveal substantial pedagogical innovation, methodological diversification, and an increasing integration of digital ecosystems, while also drawing attention to areas where further development is both necessary and promising, namely equity, digital literacy, institutional support, and the consolidation of evidence. These insights provide a critical foundation for considering the implications of innovative teaching and learning for educational practice, policy, and future research in health education and health promotion.

## Implications for practice, policy, and research

Educators must continue to strengthen their digital and pedagogical literacies so they can integrate innovation thoughtfully and ethically, ensuring that technology supports, rather than replaces, the relational, reflective and human dimensions of teaching. At the policy level, digital transitions require sustained structural investment, including equitable access to technological resources, teacher development programmes, culturally responsive strategies and institutional safeguards that prevent the deepening of existing inequalities. Future research will benefit from greater methodological rigor, particularly through longitudinal designs, cross-national comparisons and mixed methods approaches, while also addressing urgent ethical questions related to AI integration, such as bias, privacy, transparency and academic integrity. Finally, the participation of 292 authors from multiple regions underscores the value of international knowledge exchange; expanding global collaboration, especially through the inclusion of under-represented regions, will be essential for promoting equitable and sustainable progress in health education.

## Conclusion

This Research Topic brings together a diverse set of contributions that collectively illuminate the future of health education and health promotion. While the most-viewed articles highlight strong community interest in AI, digital learning environments, and data-rich ecosystems, the full Topic underscores that innovation must go far beyond technological adoption. It must be grounded in equity, ethics, rigorous evaluation, humanistic values, and a commitment to learner wellbeing.

As health systems worldwide confront demographic shifts, rising chronic disease burdens, cultural diversity, and digital complexity, educational innovation becomes an essential catalyst for preparing future professionals and empowering communities. We hope this editorial fosters continued dialogue, critical reflection, and collaborative inquiry across disciplines and countries, ensuring that innovation translates into more equitable, compassionate, and impactful learning.
